# Current Advances towards 4-Hydroxybutyrate Containing Polyhydroxyalkanoates Production for Biomedical Applications

**DOI:** 10.3390/molecules26237244

**Published:** 2021-11-29

**Authors:** Ruchira Mitra, Hua Xiang, Jing Han

**Affiliations:** 1 State Key Laboratory of Microbial Resources, Institute of Microbiology, Chinese Academy of Sciences, Beijing 100101, China; mitra.ruchira@yahoo.com; 2 International College, University of Chinese Academy of Sciences, Beijing 100049, China; 3 College of Life Science, University of Chinese Academy of Sciences, Beijing 100049, China

**Keywords:** microbial synthesis, polyhydroxyalkanoates, 4HB-containing PHA, elastic polymer

## Abstract

Polyhydroxyalkanoates (PHA) are polyesters having high promise in biomedical applications. Among different types of PHA, poly-4-hydroxybutyrate (P4HB) is the only polymer that has received FDA approval for medical applications. However, most PHA producing microorganisms lack the ability to synthesize P4HB or PHA comprising 4-hydroxybutyrate (4HB) monomer due to their absence of a 4HB monomer supplying pathway. Thus, most microorganisms require supplementation of 4HB precursors to synthesize 4HB polymers. However, usage of 4HB precursors incurs additional production cost. Therefore, researchers have adopted strategies to reduce the cost, such as utilizing low-cost substrate as well as constructing 4HB monomer supplying pathways in microorganisms. We herein summarize the biomedical applications of P4HB, the natural producers of 4HB polymer, and the various strategies that have been applied in producing 4HB polymers in non-4HB producing microorganisms. It is expected that the readers would gain a vivid idea on the different strategic developments in the field of 4HB polymer production.

## 1. Introduction

As the global concerns about environmental sustainability is rising, bioplastics are gaining increasing importance as a viable alternative to petrochemical-based plastics [1]. Bioplastics are environmentally friendly, biodegradable materials derived from renewable sources. Polyhydroxyalkanoates (PHA) are polyesters usually produced by various bacteria and archaea as carbon and energy storage under unbalanced growth conditions of limiting nutrients and excess carbon supply [2]. Besides complete biodegradability in nature, the mechanical, crystal, and thermal properties of PHA are almost comparable with the petroleum counterparts [3]. Importantly, properties of PHA polymers somehow depend on their monomer composition. By changing the monomer type and composition, PHA with favorable material properties can be achieved [4]. There are at least 150 different hydroxyalkanoates that have been identified as PHA monomers [5]. Depending upon the carbon length of these monomers, PHA are classified as short-chain length (SCL-PHA, C3-C5 monomer), medium-chain length (MCL-PHA, C6-C14 monomer), and long-chain length (LCL-PHA, more than 14 carbons in monomer) [6].

SCL-PHA are the most commonly synthesized polymers by bacteria and archaea. Three monomers of SCL-PHA that have received considerable attention are 3-hydroxybutyrate (3HB), 3-hydroxyvalerate (3HV), and 4-hydroxybutyrate (4HB). Polyhydroxybutyrate (PHB), the homopolymer of 3HB, is the most widely produced PHA. PHB is brittle and highly crystalline in nature [7]. It has a high melting point and narrow processing window that makes its industrial processing difficult [8]. Interestingly, the copolymer of 3HB and 3HV, poly(3-hydroxybutyrate-*co*-3-hydroxyvalerate) (PHBV) shows enhanced mechanical properties like tensile strength, toughness, and Young’s modulus, and also reduced melting point [9]. The polymer becomes less crystalline that improves its processing ability. Notably, the homopolymer of 4HB, poly-4-hydroxybutyrate (P4HB), features superior properties and it is the only PHA that has received FDA approval for medical applications since 2007 [10].

Microbial synthesis of 4HB-containing PHA is one of the hot topics in PHA field. Researchers are continuously engrossed in developing various strategies to realize P4HB synthesis in microbes, and to achieve 4HB-containing polymers with tailor-made 4HB content. The approaches range from precursor supplementation to metabolic engineering of microbial strains. The present review discusses the various research techniques of P4HB or its copolymer synthesis. Readers shall gain an understanding as to how the techniques have evolved to enhance the production of the polymer. In this review, we have first discussed the medical applications of P4HB. In the next section, we have presented the natural producers of 4HB polymer and also the 4HB synthesis pathway. Following this, we have categorically divided the different approaches of obtaining 4HB polymers in non-natural 4HB producers. These include precursor supplementation, utilization of low-cost substrates, optimization of cultivation parameters, and finally metabolic engineering to construct 4HB synthesis pathways.

## 2. Medical Application of P4HB

P4HB is a strong, pliable, and resorbable thermoplastic biomaterial [11]. P4HB is extremely elastic, and its tensile properties are almost comparable to the ultra-high-molecular-weight polyethylene [12]. It is highly ductile in nature and has an elongation to break of 1000%, indicating that it can be stretched ten times to its original size before breaking [13]. P4HB has excellent thermal processability and can be fairly molded into various structures including fibers, films, tubes, and microspheres [10]. Tepha’s TephaFLEX^®^ is a P4HB biopolymer. Interestingly, the monofilament sutures fabricated using this P4HB polymer was 35% stronger than the synthetic polydioxanone suture and 19% stronger than the polypropylene suture [14]. Therefore, P4HB fibers have been used as the starting material for medical textile products like patches, grafts, surgical meshes, etc. [15]. Phasix™ mesh is a resorbable biosynthetic mesh prepared from P4HB [16]. Interestingly, it provided long-term mechanical strength during hernia repair surgery [17]. This might prevent further surgical complications and reduce the rate of hernia recurrence. It has also been observed that the P4HB-based surgical mesh reduced the chances of post-operative surgical site infections [18]. Thus, Phasix™ may become a treatment option for hernia repair. Furthermore, P4HB are being successfully implemented in tissue engineering. Compared to polyglycolic acid (PGA) mesh, P4HB scaffolds showed prolonged degradation and also promoted tissue regeneration [19]. P4HB-based scaffold for trileaflet heart valves, vascular grafts, and artery augmentation, and P4HB pelvic floor implants are already under consideration [14,20]. Moreover, degradation of P4HB does not produce acidic by-products at wound sites and elicits mild inflammatory response during wound healing [21]. Due to its unique characteristics, P4HB is recognized as a promising biodegradable and biocompatible biopolymer having immense potentials in biomedical engineering. However, high production cost is still an obvious challenge faced by PHA research. To commercialize the use of P4HB and make it available for use in daily lives, it is important to curb down the production cost of P4HB.

## 3. Microbial Synthesis of P4HB

Several methods have been adopted to synthesize P4HB chemically. However, due to the involvement of various organometallic or metal complex catalysts such as Lewis acid, lanthanum amide, distannoxane complex, and titanium, chemical synthesis of P4HB is not suitable for biomedical applications [10]. Thus, P4HB synthesis through fermentation processes is the most desirable approach. Unlike PHB, a major drawback of microbial P4HB production is that the naturally occurring P4HB-producing microbes are very limited. Most microbes can’t produce the P4HB homopolymer rather they tend to incorporate 3HB and/or 3HV monomers, resulting in the formation of 4HB copolymers or terpolymers. Both 4HB *co*- and terpolymers have novel and fascinating properties that are favoured over PHB and PHBV. Therefore, incorporating different proportions of 3HB, 3HV, and 4HB monomers to yield tailor-made *co*- or terpolymer with desirable characteristics is an interesting research area. However, the fine balance between the monomeric units is critical. Sometimes, a high 3HB and 3HV content overshadows the benefits of 4HB content [22]. Therefore, optimizing the monomer composition is often challenging and requires extensive investigation. Taken together, a high 4HB content is always preferable and thus, continuous efforts are being made to synthesize PHA polymers having a high 4HB molar fraction. In addition, obtaining 4HB polymers at reduced cost is also a challenge faced by researchers in this field. In the following sections, different techniques like precursor supplementation, utilization of low-cost substrate, optimizing the feeding strategies and process parameters, and heterologous pathway construction to synthesize 4HB-containing polymers have been focused. Table 1 summarizes the production of 4HB polymer by different microorganisms.

### 3.1. Natural 4HB Polymer Producers and 4HB-CoA Supplying Pathway

*Hydrogenophaga pseudoflava* is one of the few known microbes capable of producing 4HB polymer from structurally unrelated carbon sources. Using L-arabinose as the substrate, the strain synthesized the terpolymer P(3HB-*co*-1 mol% 3HV-*co*-5 mol% 4HB) with a PHA content of 45.3 wt% (CDW) [23]. Thus, *H. pseudoflava* is a potentially important strain that should be further investigated to understand the metabolic pathway involved in 4HB polymer synthesis from unrelated carbon sources.

**Figure 1:**
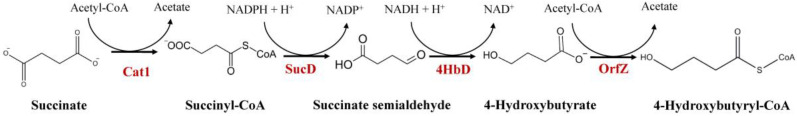
Naturally occurring 4HB-CoA supplying pathway in *Clostridium kluyveri*. Cat1, succinyl-CoA:CoA transferase; SucD, succinate semialdehyde dehydrogenase; 4HbD, 4-hydroxybutyrate dehydrogenase; OrfZ, 4HB-CoA: CoA transferase.

Synthesis of 4HB polymer requires generation of the 4HB-CoA monomer. Using structurally unrelated carbon sources like glucose, 4HB-CoA is synthesized from succinyl-CoA [13]. The synthesis of 4HB monomer from the succinate degradation pathway was characterized in *Clostridium kluyveri* [24]. In this pathway, succinate is converted to succinyl-CoA by succinyl-CoA: CoA transferase. The latter is then converted into succinate semialdehyde (SSA) by SSA dehydrogenase (Figure 1). The SSA is reduced to 4HB by 4HB dehydrogenase. 4HB-CoA: CoA transferase converts 4HB to 4HB-CoA. However, this strain is not able to synthesize 4HB polymer rather 4-hydroxybutyryl-CoA dehydratase/vinylacetyl-CoA Δ^3^-Δ^2^-isomerase converts 4HB-CoA to crotonyl-CoA. For synthesis of 4HB polymer, under the action of PHA synthase, 4HB-CoA is incorporated into the polymer chain instead of channelizing it to other pathways. Most often, the genes involved in the 4HB-CoA generation pathway is absent or suppressed in microbes and thus, they are incapable of synthesizing 4HB polymer from unrelated carbon sources. This necessitates supplementation of structurally related 4HB precursors for the incorporation of 4HB monomers into polymer chain.

### 3.2. Precursor Supplementation to Yield 4HB Copolymers

Precursor supplementation is the most important prerequisite for synthesis of 4HB polymers in many microbes. The commonly used 4HB precursors include γ-butyrolactone, 1,4-butanediol, and 1,6-hexanediol. These precursors are metabolized to form 4HB, and then 4HB is converted to 4HB-CoA by 4HB-CoA: CoA transferase.

Studies on 4HB polymers dates back to late 1980s. For the first time, the synthesis of 4HB-containing PHA polymer was observed in *Cupriavidus necator* (also known as *Ralstonia eutropha* or *Alcaligenes eutrophus*) [51]. The strain produced PHB in the presence of carbon sources like fructose or butyric acid. However, co-feeding of γ-butyrolactone resulted in the synthesis of P(3HB-*co*-4HB) copolymer. γ-butyrolactone alone increased the 4HB content to 21 mol% in the polymer. Co-feeding γ-butyrolactone with fructose decreased the 4HB content to 4 mol% as fructose led to the formation of more 3HB-CoA moieties. Strikingly, the mixture of γ-butyrolactone and butyric acid yielded a higher 4HB molar fraction (24 mol%), indicating that butyric acid also contributed to the formation of 4HB-CoA monomer [51]. As the 4HB molar fraction increased in the copolymer, its crystallinity decreased and elongation to break increased. At 3 mol% of 4HB, crystallinity of the copolymer was 55% and elongation to break was 45%. Interestingly at 16 mol%, the crystallinity decreased to 45% and the elongation to break increased to 444%. Finally, due to the parameters’ change, the physical properties of the polymer improved [51]. P(3HB-*co*-4HB) copolymer with a high 4HB molar fraction is more preferable for biomedical applications. Following this, several precursor feeding strategies have been explored for achieving a higher 4HB content in the PHA polymers. For instance, co-feeding citrate and ammonium sulphate with 4-hydroxybutyric acid led to the synthesis of P4HB homopolymer in *C. necator*. Unfortunately, the P4HB content was very low (≈ 2 wt% CDW) [27]. Instead, the addition of *n*-alkanoic acid like propionic acid in the presence of ammonium sulphate and 4-hydroxybutyric acid increased the content of P4HB homopolymer to 34 wt% (CDW) in the strain [28]. *Comamonas acidovorans* produced P(3HB-*co*-4HB) random copolymer from 4-hydroxybutyric acid and glucose or 3-hydroxybutyric acid [52]. Furthermore, it produced a terpolymer P(3HB-*co*-3HV-*co*-4HB) from 1,4-butanediol and pentanol [53]. Interestingly, the strain produced 28 wt% (CDW) of P4HB homopolymer using 1,4-butanediol or 4-hydroxybutyric acid as the sole carbon source [27]. *Alcaligenes latus* is a natural PHB producer [54]. In the presence of sucrose and γ-butyrolactone, the strain produced P(3HB-*co*-4HB) [55]. The conversion efficacy of γ-butyrolactone to 4HB monomer was almost 60%. Increasing the γ-butyrolactone concentration from 0.1 to 2.0 g/L, the 4HB content in polymers varied from 4 to 45 mol%. *H. pseudoflava* produces PHB from glucose [23]. Supplementation of γ-butyrolactone resulted in 4HB incorporation up to 66 mol%. Moreover, it also utilized ε-caprolactone as the 4HB precursor when co-fed with glucose to incorporate 20 mol% of 4HB monomer. All these early studies established the importance of precursor supplementation for improving 4HB fraction in 4HB-containing polymers.

Since 2000, more and more microbial strains have been explored to synthesize 4HB polymers using different composition of 4HB precursor mixture. Combination of 1,4-butanediol and γ-butyrolactone produced P(3HB-*co*-4HB) with a 4HB content as high as 84 mol% in *Cupriavidus malaysiensis* [33]. *C. malaysiensis* produced 70 wt% P(3HB-*co*-4HB) with 4HB fractions ranging from 31 mol% to 41 mol% by using a mixture of 1,4-butanediol and 1,6-hexanediol [34]. Co-feeding γ-butyrolactone and 1,6-hexanediol yielded a polymer with a higher 4HB molar fraction (55 mol%) in this strain [56]. Using carbon sources such as propionic acid or butyric acid with 4-hydroxybutyric acid in the presence of α-amino acids, *C. necator* produced P(3HB-*co*-4HB) with 72–86 mol% 4HB [57]. Propionic acid has a stimulating effect on 4HB polymer synthesis. The recombinant *E. coli*, engineered to synthesize P4HB, produced a higher content of P4HB when propionic acid was supplied along with glycerol. The content increased from 30 to 80 wt% (CDW) when propionic acid concentration increased from 0 to 2 g/L [43].

### 3.3. Microbial Synthesis of 4HB Terpolymer

Studies on the synthesis of 4HB terpolymer is considerably limited. Till now, few microbes namely *C. necator*, *C. malaysiensis*, *Aneurinibacillus* sp. H1, *H. pseudoflava*, and haloarchaeon *Haloferax mediterranei* have been reported to synthesize terpolymer P(3HB-*co*-3HV-*co*-4HB). Using 4-hydroxybutyric acid or γ-butyrolactone as 4HB precursor and propionic acid as 3HV precursor along with glucose, *C. necator* produced several types of the terpolymer P(3HB-*co*-3HV-*co*-4HB) [58]. However, the terpolymer comprising approximately 10 mol% of 3HV and 4HB each showed better mechanical properties than P(3HB-*co*-30 mol% 3HV). Changing the carbon source type and ratio further changed the 4HB molar fraction in its terpolymer by using *C. necator*. For example, the supplementation of 4-hydroxybutyric acid to the medium containing fructose or butyric acid, and valerate resulted in the production of terpolymer containing 4HB up to 84 mol% [31]. Moreover, the 4HB content further increased to 93 mol% with increasing cultivation time. Replacing the 4HB precursor with 1,4-butanediol, however, reduced the 4HB content to almost 60 mol% in this strain. A thermophilic gram-positive bacterium *Aneurinibacillus* sp. H1 accumulated PHB when cultured on glucose or glycerol. However, when supplemented with 1,4-butanediol, the strain produced P(3HB-*co*-4HB) with 4HB molar fraction up to 90 mol%. Moreover, by cofeeding valerate, it was able to synthesize terpolymer P(3HB-*co*-33 mol% 3HV-*co*-54 mol% 4HB) [25].

Ramachandran et al. (2011) conducted a detailed investigation to obtain a superior terpolymer in *C. malaysiensis* by varying the composition of 4HB and 3HV precursors [22]. A combination of 1,4-butanediol, 1-pentanol, and palmitic acid led to the synthesis of P(3HB-*co*-4 mol% 3HV-*co*-33 mol% 4HB) showing high Young’s modulus (101 MPa) and elongation to break up to 937%. Furthermore, P(3HB-*co*-18 mol% 3HV-*co*-33 mol% 4HB) was synthesized by slightly increasing the concentration of 1-pentanol in the same substrate mixture and the polymer showed lower Young’s modulus (34 MPa) and reduced elongation to break (554%). However, palmitic acid had a negative impact on the cell growth and thus reduced the PHA yield (≈ 0.8 g/L). In contrast, oleic acid supported cell growth and improved PHA production to 3.3 g/L by generating more acetyl-CoA via the β-oxidation pathway. Oleic acid favoured 3HB incorporation and hence reduced the 4HB content to almost 24 mol% in the terpolymer [22,59]. Combining oleic acid and γ-butyrolactone with 1-pentanol, a high terpolymer content up to 81 wt% (CDW) was obtained [35]. Dissolved oxygen (DO)-stat fed-batch fermentation using the same substrate mixture was employed to improve the yield of terpolymer in *C. malaysiensis* [59]. Three different fed-batch fermentation produced P(3HB-*co*-35 mol% 3HV-*co*-16 mol% 4HB), P(3HB-*co*-16 mol% 3HV-*co*-22 mol% 4HB), and P(3HB-*co*-10 mol% 3HV-*co*-7mol % 4HB) at a yield of 4.2, 8.2, and 16.7 g/L, respectively. Both *H. pseudoflava* and *H. mediterranei* synthesized 4HB-containing terpolymer by utilizing low-cost substrates such as cheese whey and crude glycerol as discussed in the following section.

### 3.4. Utilization of Low-Cost Substrate for 4HB Polymer Synthesis

Synthesis of PHA is cost-intensive mainly due to the use of high-priced raw materials. Therefore, utilization of low-cost substrates for PHA synthesis has been a prominent research area. Usage of low-cost substrates such as food waste and agro-industrial wastes would not only contribute in curbing down the production cost, but would be a step towards waste valorization.

*C. necator* is able to synthesize 4HB polymer by using low-cost substrates including spent palm oil and waste glycerol. Using spent palm oil left after frying activities and 1,4-butanediol, the strain produced P(3HB-*co*-15 mol% 4HB) [60]. Similarly, by using waste glycerol from biodiesel plant and γ-butyrolactone, P(3HB-*co*-4HB) with a 4HB incorporation up to 21.5 mol% was achieved [29]. Moreover, co-feeding γ-butyrolactone with soybean oil, *C. necator* produced P(3HB-*co*-4HB) with 4HB molar fractions ranging from 6 to 10 mol% [30]. Glycerine pitch is a routine waste generated from oleochemical industries. *Cupriavidus* sp. USMAHM13 produced P(3HB-*co*-4HB) with a 4HB molar fraction of 43 mol% by using glycerine pitch and 1,4-butanediol [36]. Wheat straw hydrolysates serve as an excellent substrate for PHA production in *Burkholderia sacchari*. Co-feeding γ-butyrolactone with wheat straw hydrolysates, the strain accumulated P(3HB-*co*-5 mol% 4HB) [26]. Using glucose and γ-butyrolactone, the recombinant *H. bluephagenesis*, capable of synthesizing 4HB polymer, produced 81.4 g/L CDW containing 73.8% P(3HB-*co*-11.6 mol% 4HB) [48]. When glucose was replaced with waste gluconate obtained from corn starch processing, the strain produced 68.1 g/L CDW containing 70.6% P(3HB-*co*-12.4 mol% 4HB). Not only the PHA yield was comparable to that obtained from glucose, but use of waste gluconate would reduce the raw material cost.

Synthesis of terpolymer using low-cost substrates have also been reported in few microbial species. In the presence of waste glycerol, γ-butyrolactone and propionic acid, *C. necator* produced P(3HB-*co*-4HB-*co*-3HV) with 4HB molar fraction ranging from 24.8 to 43.6 mL% and 3HV% from 5.6 to 9.8 mol% [29]. *H. pseudoflava* was able to synthesize the terpolymer P(3HB-*co*-2.2 mol% 3HV-*co*-18.4 mol% 4HB) using cheese whey as the sole carbon source [39]. In the Archaea domain, haloarchaeon *H. mediterranei* was capable of incorporating the 4HB monomers by using 4HB precursors. *H. mediterranei* is a natural producer of PHBV from structurally unrelated carbon sources such as glucose and chitin [61,62]. This strain is capable of producing 16.2 g/L of P (3HB-*co*-10 mol% 3HV) by utilizing crude glycerol from biodiesel production as the sole carbon source [37]. Supplying γ-butyrolactone with the crude glycerol led to the synthesis of P(3HB-*co*-12 mol% 3HV-*co*-5 mol% 4HB) with a yield of 11.1 g/L. Similarly, in the presence of whey sugars as the sole carbon source, the strain synthesized 72.8 wt% of P(3HB-*co*-6 mol% 3HV) [38]. The addition of valerate and γ-butyrolactone along with whey sugars resulted in the synthesis of 87.5 wt% of P(3HB-*co*-21.8 mol% 3HV-*co*-5.1 mol% 4HB). Notably, PHA synthesis using *H. mediterranei* has some inherent advantages over bacteria. Fermentation of *H. mediterranei* does not involve stringent sterilization procedures because its high salinity requirement reduces chances of contamination [63]. Upon exposure to normal water, *H. mediterranei* cells undergo cell lysis which eases PHA recovery. Additionally, *H. mediterranei* has a tractable genetic system that provides the opportunity to improve 4HB-containing terpolymer synthesis with the aid of metabolic engineering.

### 3.5. Cultivation Parameters Affecting 4HB Polymer Synthesis

Other than the type of carbon source, polymer synthesis is greatly affected by culture conditions and cultivation modes. Vigneswari et al., 2009 reported that cell concentration, phosphate ratio, and aeration in a culture influenced the P(3HB-*co*-4HB) synthesis as well as the 4HB molar fraction in *C. malaysiensis* [64]. With increasing cell concentration in the medium from 0.33 to 1.67 g/L, the P(3HB-*co*-4HB) yield increased from 0.58 to 1.25 g/L and the 4HB molar fraction increased from 33 to 52 mol% in this strain. When the phosphate ratio increased from 0 to 2.45, the yield of P(3HB-*co*-4HB) increased from 0.21 to 2.49 g/L. Moreover, the 4HB molar fraction in polymers ranged between 29–54 mol%. Above a ratio of 2.45, the polymer yield was slightly reduced. It was suggested that after a certain limit, phosphate concentration had a negative effect on polymer accumulation. Besides, culture aeration affected the P(3HB-*co*-4HB) yield and 4HB incorporation. Culture aeration decreased by increasing the culture volume at a fixed flask volume. With decreasing culture aeration, the polymer yield as well as 4HB molar fraction increased. Increasing the culture volume from 30 to 80 mL in a 250-mL flask, the 4HB incorporation increased from 23 to 75 mol%. Thus, oxygen limitation facilitated 4HB incorporation. In fact, P(3HB-*co*-4HB) production in 30-L reactor showed a higher 4HB incorporation (40 mol%) at lower agitation speed (150 rpm) [65]. At higher agitation speeds (200 rpm and 250 rpm), 4HB incorporation was ~28 mol%. In further studies, employing fed-batch cultivation strategies increased the 4HB molar fraction in an engineered *C. malaysiensis* [66]. Instead of feeding only carbon source, a pulse feed of carbon and nitrogen gave better performances. Twice pulse feed of carbon and nitrogen gave the best output, yielding 92 wt% (CDW) of P(3HB-*co*-99 mol% 4HB) with a PHA yield of 46 g/L. Moreover, maintaining a minimal level of nitrogen supplementation that is sufficient enough to support the cell growth required for polymer synthesis is recommended. Implementation of two-stage cultivation of *C. malaysiensis* is a promising approach to improve 4HB molar fraction in P(3HB-*co*-3HV-*co*-4HB) and enhance the PHA production simultaneously. For instance, using γ-butyrolactone and 1-pentanol in two-stage cultivation, 4HB incorporation up to 14 mol% and terpolymer concentration of 2.5 g/L was achieved [67]. When the culture aeration was reduced, the 4HB molar fraction further increased to 24 mol%. In a further study, two-stage cultivation using glycerol with 1,4-butanediol in the first stage, and 1,4-butanediol with valerate in the second stage, 4.14 g/L of P(3HB-*co*-17.86 mol% 3HV-*co*-16.46 mol% 4HB) was obtained by using the same microbe [68].

The 4HB polymer production in *C. necator* was improved by employing DO-stat strategy for feeding the mixture of fructose and γ-butyrolactone [69]. In flask experiments, a 4HB molar fraction up to 27.4 mol% was obtained by using fructose and γ–butyrolactone but the higher concentrations of both the substrates inhibited cell growth. Additionally, in the presence of a stimulator like propionate or acetate, a 4HB molar fraction of 53 mol% was achieved but the PHA production (0.27 g/L) was greatly reduced. However, the fed batch strategy improved polymer yield as PHA yield in the range of 14–24.4 g/L was obtained by varying the fructose to γ–butyrolactone ratio in the feeding solution. However, the 4HB molar fraction was less than the shake flask experiments and it ranged between 25.2 and 1.64 mol%. Similarly, the substrate ratio affected the 4HB molar fraction in P(3HB-*co*-4HB) in *C. necator* A-04 [70]. Using the mixture of γ-hydroxybutyric acid and butyric acid as carbon source and ammonium sulphate as nitrogen source, different monomer compositions were obtained by changing the C/N ratio. At a high C/N ratio, i.e., nitrogen deficient condition, 3HB incorporation was favoured whereas when the C/N ratio was low (i.e., nitrogen sufficient condition), 4HB incorporation was preferred. A C/N ratio of 4 resulted in a 4HB molar fraction of 70 mol% in *C. necator* A-04. The 4HB molar fraction in P(3HB-*co*-4HB) synthesized by *Burkholderia sacchari* was improved by finely tuning the fed batch strategy [71]. The γ–butyrolactone feeding was optimized to obtain the best outcome. A γ–butyrolactone pulse of 10 g/L added at the initial polymer accumulation phase followed by its constant addition at a rate similar to its consumption ensured the maximal incorporation of 7.1 mol% 4HB. Taken together, synthesis of 4HB polymer containing desirable proportion of 4HB is directly related to the composition and concentration of the substrate mixture and the culture parameters.

## 4. Metabolic Engineering to Synthesize 4HB Polymers

Supplementation of 4HB precursor for synthesis of 4HB-containing polymer incurs additional production cost. Moreover, some microbes including *E. coli* and *Halomonas bluephagenesis* TD01 are even incapable of utilizing 4HB precursors as carbon source. Thus, metabolic engineering is a better solution to enable 4HB polymer synthesis in such microbes. Pathway engineering that includes heterologous 4HB synthesis pathway de novo construction has successfully allowed 4HB polymer synthesis in several microbes.

### 4.1. Construction of Recombinant E. coli to Synthesize 4HB Polymer

**Figure 2:**
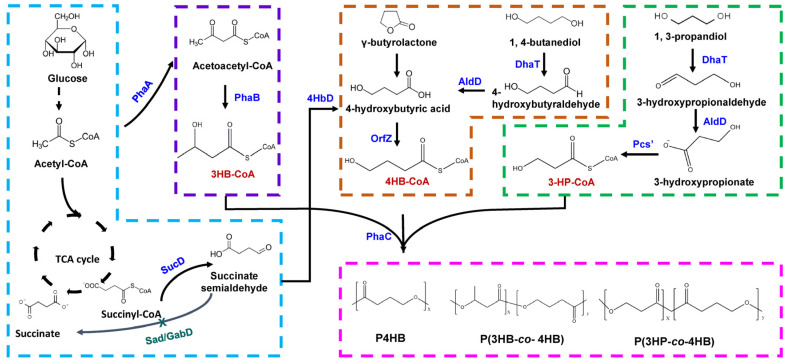
Different metabolic engineering strategies to synthesize 4HB-containing PHA in *Escherichia coli*. When using glucose as the sole substrate, it is converted to succinyl-CoA via glycolytic pathway and TCA cycle. Succinyl-CoA is converted to succinate semialdehyde by SucD. Deletion of native *sad*/*gabD* genes prevents the conversion of succinate semialdehyde to succinate. Then, succinate semialdehyde is converted to 4-hydroxybutyric acid by 4HbD. When using γ-butyrolactone or 1, 4-butanediol as 4HB precursor, they are converted to 4-hydroxybutyric acid. 4-hydroxybutyric acid is then converted to 4HB-CoA by OrfZ. 3HB-CoA is derived from acetyl-CoA under the action of PhaA and PhaB. Finally, P4HB or P(3HB-*co*-4HB) is synthesized under the action of PhaC. For the synthesis of P (3HP-*co*-4HB), 3HP-CoA is derived from 1,3-propandiol. Under the action of PhaC, 3HP-CoA and 4HB-CoA is polymerized to form P (3HP-*co*-4HB). SucD, succinate semialdehyde dehydrogenase; Sad and GabD, succinate semialdehyde dehydrogenase; PhaA, β-ketothiolase; PhaB, acetoacetyl-CoA reductase; PhaC, PHA synthase; 4hbD, 4-hydroxybutyrate dehydrogenase; OrfZ, CoA transferase; DhaT, 1,3-propanediol dehydrogenase; AldD, aldehyde dehydrogenase; Pcs’, ACS domain of propionyl-CoA synthase.

Wild type *E. coli* is incapable of synthesizing PHA due to the lack of *pha* synthesis genes. However, PHB synthesis in *E. coli* has been made possible by pathway engineering. Similarly, recombinant *E. coli* was able to synthesize 4HB polymer when 4HB synthesis pathway was established in this strain (Figure 2). The entire PHA synthase gene (*phaC*) and 878 of 1179-bp of the 5′ region of the β-ketothiolase gene (*phaA*) from *C. necator* plus the entire 4HB-CoA transferase gene (*orfZ*) from *Clostridium kluyveri* were heterologously expressed in *E. coli* [72]. Supplementing M9 mineral salt medium with 4-hydroxybutyric acid and glucose, the recombinant *E. coli* synthesized ~80 wt% (CDW) P4HB homopolymer under oxygen-deficient condition. Even when replacing 4-hydroxybutyric acid with γ-butyrolactone, the *E. coli* strain synthesized 16.1 wt% (CDW) P4HB homopolymer. Another study by Valentin and Dennis (1997) reported that the *phaCAB* gene cluster (encoding PHA synthase, β-ketothiolase, and acetoacetyl-CoA reductase, respectively) from *C. necator* and *orfZ-sucD-4hbD* (encoding CoA transferase, succinate semialdehyde dehydrogenase, and 4-hydroxybutyrate dehydrogenase, respectively) from *C. kluyveri* were co-expressed in *E. coli* [40]. Using glucose as the sole carbon source, the engineered *E. coli* accumulated 50 wt% (CDW) P(3HB-*co*-2.8 mol% 4HB). It was possible that due to the toxicity of succinate semialdehyde, formed from succinyl-CoA under the action of *sucD*, succinate semialdehyde was degraded to succinate by its native succinate dehydrogenase in *E. coli*. This reduced the metabolic flux of glucose for 4HB-CoA generation. Thus, in another study by Li et al. (2010), *sad* and *gabD* genes were both knocked out to avert the degradation of succinate semialdehyde [41]. Interestingly, the resulting *E. coli* strain produced 65.5 wt% (CDW) P(3HB-*co*-11.1 mol% 4HB) using glucose as the sole carbon source. Furthermore, to promote the succinyl-CoA supplying pathway, TCA cycle intermediates such as 2-oxoglutarate, oxaloacetate, and citrate were supplemented in the media which increased the 4HB mol% to ~22 mol%, 16.3 mol%, and 20.3 mol%, respectively. Keeping the same genetic background, four phasin encoding genes (*phaP1*, *phaP2*, *phaP3*, and *phaP4*) from *C. necator* were individually expressed in the recombinant *E. coli* [13]. Interestingly, in the shake flasks containing LB medium supplemented with glucose, *E. coli* with no *phaP* gene synthesized only 12.13 wt% (CDW) P4HB whereas individual *phaP* expression increased the content to ~22 wt% (CDW) with *phaP2* or *phaP4*; 32.35 wt% (CDW) with *phaP3,* and 35.39 wt% (CDW) with *phaP1*. Furthermore, in fed-batch fermentation, ~68 wt% (CDW) P4HB was produced by the recombinant *E. coli* with *phaP1* expression. In another study, the 4-hydroxybutyric acid was first produced from 1, 4-butanediol by a recombinant *Aeromonas hydrophila* strain harbouring *dhaT* and *aldD* (encoding 1, 3-propanediol dehydrogenase and aldehyde dehydrogenase, respectively) from *Pseudomonas putida* KT2442 [42]. Then the 4HB-containing fermentation broth was used to synthesize P4HB in the recombinant *E. coli*. *dhaT* and *aldD* from *P. putida* KT2442, *orfZ* from *C. kluyveri*, and *phaC1* from *C. necator* were simultaneously expressed in *E. coli*. The resultant recombinant strain produced 2 g/L CDW containing 83 wt% of P(4HB) when using glucose and the 4-hydroxybutyric acid as the carbon source. The presence of *dhaT* and *aldD* in *E. coli* helped in converting any residual 1,4-butanediol to 4HB, thus, increasing the supply of 4HB for P4HB synthesis.

Another novel copolymer of 4HB and 3-hydroxypropionic acid (3HP) has been synthesized in *E. coli* by using metabolic engineering strategy. P3HP is known for its high tensile strength and P4HB for its elastic nature [73]. Thus, synthesis of P(3HP-*co*-4HB) possessing combined properties is a promising area in biomaterials. Synthesis of 3HP homopolymer from 1, 3-propandiol involved *dhaT*, *aldD*, *pcs’* (encoding ACS domain of propionyl-CoA synthase that functions like 3-hydroxypropanoic acid CoA ligase), and *phaC* genes [74]. Importantly, 4HB and 3HP are structurally similar and hence it was likely that the genes involved in the conversion of 3HP to 3HP-CoA might be also functional in the conversion of 1, 4-butanediol to 4HB-CoA. Therefore, *dhaT* and *aldD* from *P. putida* KT2442, *pcs’* from *Chloroflexus aurantiacus*, *phaC1* from *C. necator* were expressed in *E. coli* [74]. When supplemented with 1.3-propanediol and 1, 4-butanediol, the poly(3-hydroxypropionate-*co*-4-hydroxybutyrate) P(3HP-*co*-4HB) copolymer up to 42 wt% (CDW) was obtained. However, the 4HB molar fraction was less than 2 mol%. In another study, a different approach was adopted that led to a higher 4HB molar fraction (30 mol%) in the copolymer [44]. The *orfZ* gene from *C. kluyveri*, *pcs’* from *C. aurantiacus*, *dhaT* and *aldD* from *P. putida* KT2442, and *phaC1* from *C. necator* were expressed in *E. coli*. In the presence of 1.3-propanediol and 1, 4-butanediol, 62.70 wt% (CDW) P (70 mol% 3HP-*co*-30 mol% 4HB) was synthesized. The thermal properties of the 4HB copolymers were improved as the melting point was reduced with the incorporation of 4HB monomers compared to P3HP. Moreover, the elongation at break of the 4HB copolymer also increased compared to P3HP. However, the tensile strength and Young’s modulus of the copolymer did not show improvements except that the copolymer containing 12 mol% 4HB showed a higher tensile strength compared to P3HP. Interestingly, the copolymer containing 67 mol% of 4HB was completely transparent unlike P3HP, thus, necessitating further investigation on the synthesis of P (3HP-*co*-4HB).

### 4.2. Engineering H. bluephagenesis TD01 for Synthesis of 4HB Polymer

**Figure 3:**
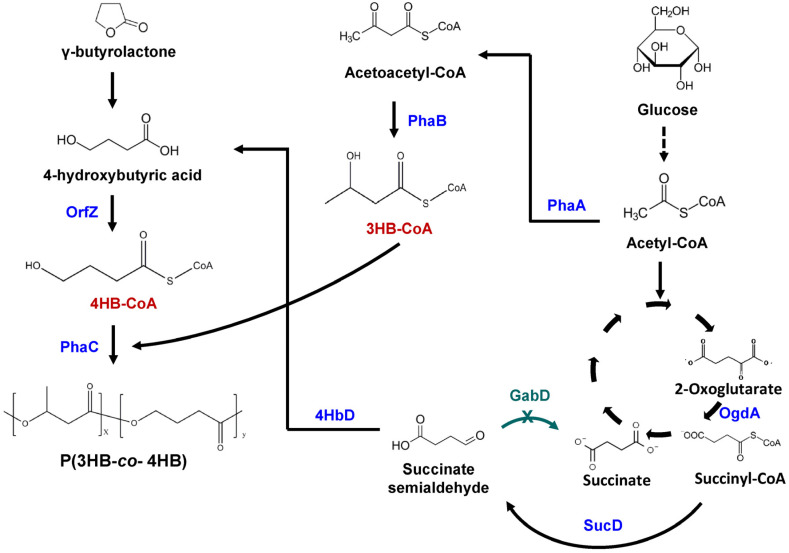
Metabolic engineering of *Halomonas bluephagenesis* TD01 to synthesize P (3HB-*co*-4HB). When using γ-butyrolactone as 4HB precursor, 4-hydroxybutyric acid is converted to 4HB-CoA by OrfZ. 3HB-CoA is derived from acetyl-CoA under the action of PhaA and PhaB. When using glucose as the sole substrate, it is converted to succinyl-CoA via glycolytic pathway and TCA cycle. OgdA catalyzes the conversion of 2-oxoglutarate to succinyl-CoA which is further converted to succinate semialdehyde by SucD. Deletion of *gabD* genes prevented the conversion of succinate semialdehyde to succinate. Next, succinate semialdehyde is converted to 4-hydroxybutyric acid by 4HbD. Under the action of PhaC, 3HB-CoA and 4HB-CoA are polymerized to form P(3HB-*co*-4HB). OgdA, 2-oxoglutarate dehydrogenase; SucD, succinate semialdehyde dehydrogenase; GabD, succinate semialdehyde dehydrogenase; 4HbD, 4-hydroxybutyrate dehydrogenase; OrfZ, CoA transferase; PhaA, β-ketothiolase; PhaB, acetoacetyl-CoA reductase; PhaC, PHA synthase.

*H. bluephagenesis* TD01 is a halophilic bacterium showing a high capability in PHA industrial production, however, it is unable to synthesize 4HB-containing PHA even in the presence of 4HB precursor. The 4HB-CoA transferase encoding gene is absent in this microbe. Hence, the *orfZ* gene from *C. kluyveri* was expressed in *H. bluephagenesis* TD01(Figure 3) [45]. Unlike *E. coli*, *H. bluephagenesis* TD01 possesses its own *pha* synthesis genes. The T7-like system was used to tune the *orfZ* expression strength. In the presence of glucose and γ-butyrolactone, the recombinant strain incorporated 11.2 mol% of 4HB in P(3HB-*co*-4HB). To ensure better genetic stability, *orfZ* was then chromosomally integrated in the genome of *H. bluephagenesis* TD01. In a 7-L fermentor, 72 g/L CDW containing 63% P(3HB-*co*-12.3 mol% 4HB) was produced in 48 h. To further scale-up the process, the fermentation was carried out in 1000-L fermentor in an open unsterile condition. After 48 h of fermentation, 82.6 g/L CDW containing 61% P(3HB-*co*-16 mol% 4HB) was achieved [45]. Furthermore, by engineering the promoter driving the expression of *orfZ* gene, 100 g/L CDW containing 80% P(3HB-*co*-11 mol% 4HB) was obtained in unsterile fed-batch fermentation [46]. Finally, to synthesize P(3HB-*co*-4HB) using only glucose, the *orfZ* gene and *ogdA* encoding 2-oxo-glutarate dehydrogenase, *sucD* and *4hbd* genes were introduced in *H. bluephagenesis* TD01 (Figure 3) [47]. This would direct succinate semialdehyde derived from 2-oxoglutarate and succinyl-CoA in the TCA cycle towards 4HB-CoA generation. Simultaneously, *gabD* genes were deleted to avoid the conversion of succinate semialdehyde to succinate [47]. The resulting strain was able to produce 26.3 g/L CDW containing 60.5% P(3HB-*co*-17 mol% 4HB) in 7-L bioreactor after 60 h of fed-batch fermentation under unsterile conditions. Moreover, by controlling the concentration of residual glucose in the culture, the 4HB fraction varied from 13.4 to 24.9 mol%. Thus, *H. bluephagenesis* TD01 holds high promise as an efficient industrial producer of P(3HB-*co*-4HB) polymer.

### 4.3. Synthesis of 4HB Polymer in Other Microbes

A PHB-leaky transposon-induced mutant strain of *C. nectar* JMP222 accumulated less PHB compared to the wild type strain. Notably, all the three PHB biosynthetic enzymes, PhaA, PhaB, and PhaC were synthesized at normal levels [75]. It was speculated that the mobilization system of the PHB-leaky mutant was affected as its PHB content dropped rapidly once the carbon source was exhausted compared to the wild-type [75]. Interestingly, the PHB-leaky mutant strain synthesized ~10 wt% (CDW) P4HB by using 1,4-butanediol or 4-hydroxybutyric acid [32]. Interestingly, overexpressing the *phaC* gene from wild-type *C. nectar* in the mutant strain increased the P4HB content to ~30 wt% (CDW). Co-expression of *dhaT* and *aldD* from *P. putida* KT2442 in *C. nectar* led to the synthesis of 6.1 g/L CDW containing 49 wt% P(3HB-*co*-13 mol% 4HB) in LB medium with fructose, 1,4-butanediol and 4-hydroxybutyric acid (produced by recombinant *A. hydrophila*) [42]. *C. malaysiensis* was able to synthesize P (3HB-*co*-4HB) with a 4HB molar fraction up to 75% using different 4HB precursors [76]. The 4HB molar fraction was further enhanced by genetic engineering. The *phaC* gene of *C. malaysiensis* USMAA1020 and *C. malaysiensis* USMAA2-4 were cloned and introduced into the wild type *C. malaysiensis* USMAA1020, respectively. This led to the P(3HB-*co*-4HB) synthesis with 93 mol% of 4HB [76]. *Burkholderia* sp. is capable of accumulating PHA and its *pha* gene cluster consists of *phaC*, *phaA*, *phaB*, and *phaR* (encoding the PHA synthesis regulator). However, it does not have the ability to produce 4HB polymer. Nevertheless, when the *phaC* gene of *Burkholderia* sp. strain JCM 15050 was expressed in the PHA-negative *C. necator* strain PHB¯4, the latter accumulated P(3HB-*co*-4HB) with a 4HB molar fraction up to 87 mol% using sodium 4-hydroxybutyrate [77]. Similarly, the heterologous expression of *phaC* from *Burkholderia contaminans* Kad1 in *C. necator* strain PHB¯4 allowed the latter to produce P(3HB-*co*-4HB) with a 4HB content up to 51 mol% from sodium 4-hydroxybutyrate [78]. The cell wall of *Bacillus* species lacks lipopolysaccharides and a novel self-destructive cell lysis system was established which eases the PHA recovery process [49,79]. This makes *Bacillus* species a promising host for PHA synthesis. Unfortunately, *Bacillus* species is incompetent of producing 4HB polymer from related carbon sources [49]. However, there are some reports showing the natural synthesis of 4HB polymer in *Bacillus cereus* from unrelated carbon sources like fructose and gluconate [80]. With the aid of metabolic engineering, 4HB polymer synthesis has been realized in *Bacillus megaterium* [49]. To synthesize P (3HB-*co*-4HB), *sucD*, *4hbD*, and *orfZ* from *C. kluyveri* under the control of xylose-inducible promoter were expressed in *B. megaterium*. Interestingly, a 4HB of 10 mol% was incorporated using glucose as the carbon source and xylose as the inducer. Hu et al. (2011) synthesized a PHA block copolymer consisting of PHB and P4HB blocks [81]. The *phaC* and *orfZ* from *C. nectar* was expressed in a β-oxidation weakened strain of *P. putida*. The resulting recombinant strain synthesized di-block copolymer in which one block was PHB and another was P4HB [81]. Cyanobacteria *Synechococcus* sp. PCC 7002 was genetically engineered to synthesize P(3HB-*co*-4HB). To realize this, the non-PHA producing wild-type strain was first engineered to synthesize PHB and then the biosynthetic pathway for synthesis of P(3HB-*co*-4HB) was constructed in the PHB-producing strain [50]. Precisely, the *phaABEC* operon encoding acetyl-CoA acetyltransferase, acetoacetyl-CoA reducatese, class III PHA synthase (PhaE subunit and PhaC subunit), respectively, from *Chlorogloeopsis fritschii* was expressed in *Synechococcus* sp. PCC 7002. The resulting strain synthesized PHB. Further deletion of the *ccmR* gene encoding LysR-type transcription factor improved the acetyl-CoA pool that improved PHB synthesis. Finally, in this engineered strain, a 4HB synthesis pathway containing the 4-hydroxybutyrate dehydrogenase (*gbd1*) and 4-hydroxybutyryl-CoA transferase (*cat2*) genes from *Porphyromonas gingivalis* and the native 2-oxoglutarate decarboxylase (*ogdA*) gene was introduced. The resulting strain accumulated 4.5 wt % (CDW) containing P(3HB-*co*-12 mol% 4HB).

## 5. Conclusion and Future Perspectives

P4HB or 4HB-containing PHA is a promising biomaterial in the biomedical field. However, its synthesis is a constraint as most microbes lack 4HB synthesis pathways. To commercialize the use of P4HB or its copolymers, it is necessary to accelerate its production. Researchers have adopted different strategies such as supplementing structurally related 4HB precursors such as 1,4-butanediol, γ-butyrolactone, and 4-hydroxybutyric acid. In addition, several research groups have employed low-cost substrates for producing 4HB polymer which would contribute to reducing the production cost of the polymer. Optimizing the cultivation parameters like feeding strategy, mode of fermentation, C/N ratio, and aeration status also help in improving the production of 4HB polymer. However, some microbes are incapable of utilizing 4HB precursors, thus, necessitating application of metabolic engineering. The 4HB synthesis pathways have been established in several microbes. Among them, *H. bluephagenesis* TD01 is an industrially proficient producer as the engineered *H. bluephagenesis* strain can produce 4HB polymer from glucose and can also be cultivated in open, and unsterile large scale fermentor. Nevertheless, more and more microbes synthesizing 4HB polymer should be explored to commercialize the use of 4HB polymer in global market. Moreover, since the use of 4HB precursor incur additional production cost, utilization of cheap sugars to synthesize 4HB polymer should be prioritized.

## Figures and Tables

**Table 1:** 4HB polymers produced by different microorganisms.

Microorganism	Genetic Modifications	Substrate	Polymer Type	Content (wt% CDW)	Reference
*Aneurinibacillus* sp. H1	--	Glycerol and 1,4-butanediol	P(3HB-*co*-84 mol% 4HB)	--	[25]
		Glycerol, valerate, and 1,4-butanediol	P(3HB-*co*-33 mol% 3HV-*co*-54 mol% 4HB)	--	[25]
*Burkholderia sacchari*	--	Wheat straw hydrolysates and γ-butyrolactone	P(3HB-*co*-5 mol% 4HB)	27	[26]
*Cupriavidus necator*	--	Citrate, ammonium sulphate, and 4-hydroxybutyric acid	P4HB	2	[27]
		Propionic acid, ammonium sulphate, and 4-hydroxybutyric acid	P4HB	34	[28]
		Waste glycerol and γ-butyrolactone	P(3HB-*co*-21.5 mol% 4HB)	17.9	[29]
		Soyabean oil and γ-butyrolactone	P(3HB-*co*-10 mol% 4HB)	80	[30]
		Waste glycerol, propionic acid, and *γ*-butyrolactone	P(3HB-*co*-6 mol% 3HV-*co*-43.6 mol% 4HB)	36.9	[29]
		Fructose, Valerate, and 1,4-butanediol	P(3HB-*co*-16 mol% 3HV-*co*-51 mol% 4HB)	30	[31]
*C. nectar* (PHB leaky mutant)	--	1,4-butanediol or 4-hydroxybutyric acid	P4HB	10	[32]
*Cupriavidus* *malaysiensis*	--	1,4-butanediol and γ-butyrolactone	P(3HB-*co*-84 mol% 4HB)	16	[33]
		1,4-butanediol and 1,6-hexanediol	P(3HB-*co*-31~41 mol% 4HB)	70	[34]
		Oleic acid, 1-pentanol, and γ-butyrolactone	P(3HB-*co*-10 mol% 3HV-*co*-9 mol% 4HB)	81	[35]
*Cupriavidus* sp. USMAHM13	--	Glycerine pitch and 1,4-butanediol	P(3HB-*co*-43 mol% 4HB)	49	[36]
*Comamonas acidovorans*	--	1,4-butanediol or 4-hydroxybutyric acid	P4HB	28	[27]
*Haloferax mediterranei*	--	Crude glycerol and γ-butyrolactone	P(3HB-*co*-10 mol% 3HV-*co*-5 mol% 4HB)	68.5	[37]
		Whey sugar, valerate and γ-butyrolactone	P(3HB-*co*-21.8 mol% 3HV-*co*-5.1 mol% 4HB)	87.5	[38]
*Hydrogenophaga pseudoflava*	--	L-arabinose	P(3HB-*co*-1 mol% 3HV-*co*-5 mol% 4HB)	45.3	[23]
		Cheese whey	P(3HB-*co*-18.4 mol%-4HB-*co*-2.2 mol%-3HV)	2.9	[39]
Recombinant *E. coli*	*phaCAB* gene cluster from *C. necator* and *orfZ-sucD-4hbD* from *C. kluyveri* were co-expressed.	Glucose	P(3HB-*co*-2.8 mol% 4HB)	50	[40]
	*phaCAB* gene cluster from *C. necator*, *orfZ-sucD-4hbD* from *C. kluyveri* were expressed; *sad* and *gabD* genes were knocked out.	Glucose	P(3HB-*co*-11.1 mol% 4HB)	65.5	[41]
	*phaCAB* gene cluster and *phaP1* gene from *C. necator*, *orfZ-sucD-4hbD* from *C. kluyveri* were expressed; *sad* and *gabD* genes were knocked out.	Glucose	P4HB	68	[13]
	*dhaT* and *aldD* from *P. putida* KT2442, *orfZ* from *C. kluyveri*, and *phaC1* from *C. necator* were expressed.	Glucose and 4-hydroxybutyric acid	P4HB	83	[42]
	*phaC* from *C. necator* and *orfZ* from *C. kluyveri* were expressed.	Xylose and 4-hydroxybutyric acid	P4HB	67	[12]
		Glycerol and propionic acid	P4HB	80	[43]
	*orfZ* from *C. kluyveri*, *pcs’* from *C. aurantiacus*, *dhaT* and *aldD* from *P. putida* KT2442, and *phaC1* from *C. necator* were expressed.	1,3-propanediol and 1,4-butanediol	P (3HP-*co*-30 mol% 4HB)	62.70	[44]
Recombinant *H. bluephagenesis*	*orfZ* gene from *C. kluyveri* was chromosomally integrated.	Glucose and γ-butyrolactone	P(3HB-*co*-16mol% 4HB)	61	[45]
	Engineering the promoter driving expression of chromosomally integrated *orfZ* gene from *C. kluyveri.*	Glucose and γ-butyrolactone	P(3HB-*co*-11mol% 4HB)	80	[46]
	*ogdA*, *sucD*, *4hbd* and *orfZ* were introduced; *gabD* genes were knocked out.	Glucose	P(3HB-*co*-17 mol% 4HB)	60.5	[47]
	*orfZ* chromosomally integrated.	Glucose and γ-butyrolactone	P(3HB-*co*-11.6 mol% 4HB)	73.8	[48]
		Waste gluconate and γ-butyrolactone	P(3HB-*co*-12.4 mol% 4HB)	70.6	[48]
Recombinant *C. necator*	*dhaT* and *aldD* from *P. putida* KT2442 were expressed.	Fructose, 1,4-butanediol and 4-hydroxybutyric acid	P(3HB-*co*-13 mol% 4HB)	49	[42]
Recombinant *Bacillus megaterium*	*sucD, 4hbD, and orfZ* from *C. necator* under xylose inducible promoter was introduced.	Glucose with xylose as inducer	P(3HB-*co*-11 mol% 4HB)	~50	[49]
Recombinant *Synechococcus* sp. PCC 7002	*phaABEC* operon from *Chlorogloeopsis fritschii*; *gbd1* and *cat2* from *Porphyromonas gingivalis* and native *ogdA* were introduced; *ccmR* gene was deleted.	Light and CO_2_	P(3HB-*co*-12 mol% 4HB)	4.5	[50]

## Data Availability

Not applicable.
